# A Rare Case of Polyserositis With Elevated Serum Cancer Antigen (CA)-125 in Systemic Lupus Erythematosus: A Case Report of Pseudo-Pseudo Meigs Syndrome

**DOI:** 10.7759/cureus.97956

**Published:** 2025-11-27

**Authors:** Abdullah K Hussein, Omar A Aljbour, Jumana A Aljbour, Husameddin A Alshaer, Salah K Alshami

**Affiliations:** 1 Faculty of Medicine, Islamic University of Gaza, Gaza, PSE; 2 Department of Internal Medicine, Gaza European Hospital, Gaza, PSE

**Keywords:** cancer antigen 125 (ca-125), lupus serositis, massive ascites, pseudo-pseudo meigs syndrome, systemic lupus erythematosus

## Abstract

Pseudo-pseudo Meigs syndrome (PPMS) is a rare condition characterized by pleural effusion, ascites, and elevated cancer antigen (CA)-125 levels in the setting of systemic lupus erythematosus (SLE) without evidence of ovarian tumors. We report a case of a 24-year-old woman with a known history of SLE for three years. Her symptoms were well-controlled with prednisolone and hydroxychloroquine. She presented with a history of shortness of breath, diffuse abdominal pain, and abdominal distension. Initial diagnostic workup revealed ascites, elevated CA-125 levels, and pleural effusion, without evidence of malignancy. A series of confirmatory tests excluded other differential diagnoses, leading to a diagnosis of PPMS. The patient was treated initially with furosemide without improvement. After confirming the diagnosis, methylprednisolone and oral prednisone were then administered with a favorable prognosis. This case contributes to the literature by highlighting PPMS as an important differential diagnosis to be considered in patients with SLE. Early recognition is crucial to minimize the need for unnecessary invasive procedures. The goal is to initiate the appropriate management as early as possible to optimize patient outcomes.

## Introduction

Systemic lupus erythematosus (SLE) is an autoimmune disease that leads to chronic inflammatory states within organ systems, resulting in heterogeneous manifestations. Serositis is seen in approximately 16% of lupus patients in the form of pleuritis and/or pericarditis [1]. Ascites occurs in only 8-11% of patients with SLE [2], and usually results from nephrotic syndrome, protein-losing enteropathy, constrictive pericarditis, or conditions unrelated to lupus. This should raise our suspicions to exclude other causes of ascites in SLE patients, particularly if it is recurrent or massive. Cancer antigen (CA)-125, a large glycosylated mucin-like glycoprotein, is a widely used tumor marker for screening of adnexal malignancies. A combination of polyserous effusions with elevated serum CA-125 often occurs with tumors. However, it is also found in tuberculosis, nephrotic syndrome, and connective tissue diseases [3]. Pseudo-pseudo Meigs syndrome (PPMS) is a combination of ascites, pleural effusion, and elevated CA-125 in SLE patients. PPMS can be diagnosed after excluding other suspected etiologies like malignancy. Treating such patients with steroids has shown favorable outcomes in several studies [2]. To our knowledge, we are reviewing the first case of PPMS in Palestine. This article was previously presented as a poster at the Asia Pacific Association of Allergy, Asthma and Clinical Immunology (APAAACI) 2024 conference in Kuala Lumpur on December 13, 2024.

## Case presentation

**Figure 1:**
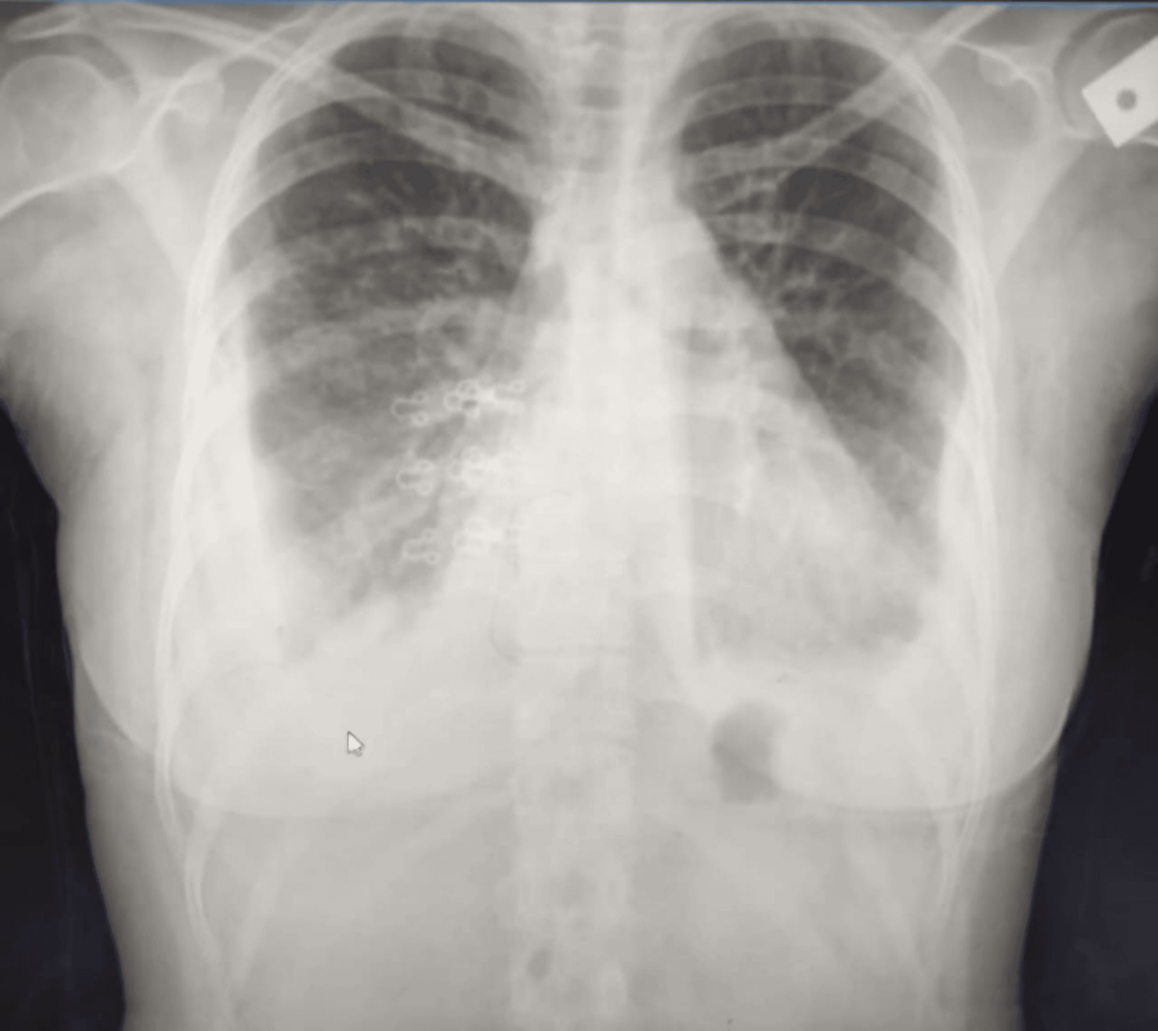
Chest X-ray revealing bilateral pleural effusion with pulmonary infiltrates

We review a case of a 24-year-old woman diagnosed with SLE in 2019. The patient was treated with prednisolone 5 mg twice daily and hydroxychloroquine (Plaquenil) 200 mg twice daily with a controlled disease course. Three years later, the patient presented with shortness of breath, diffuse abdominal pain, and abdominal distension. Physical examination revealed decreased intensity of breath sounds bilaterally, tenderness all over the abdomen, and shifting dullness. A chest X-ray revealed bilateral pleural effusion (Figure 1).

**Figure 2:**
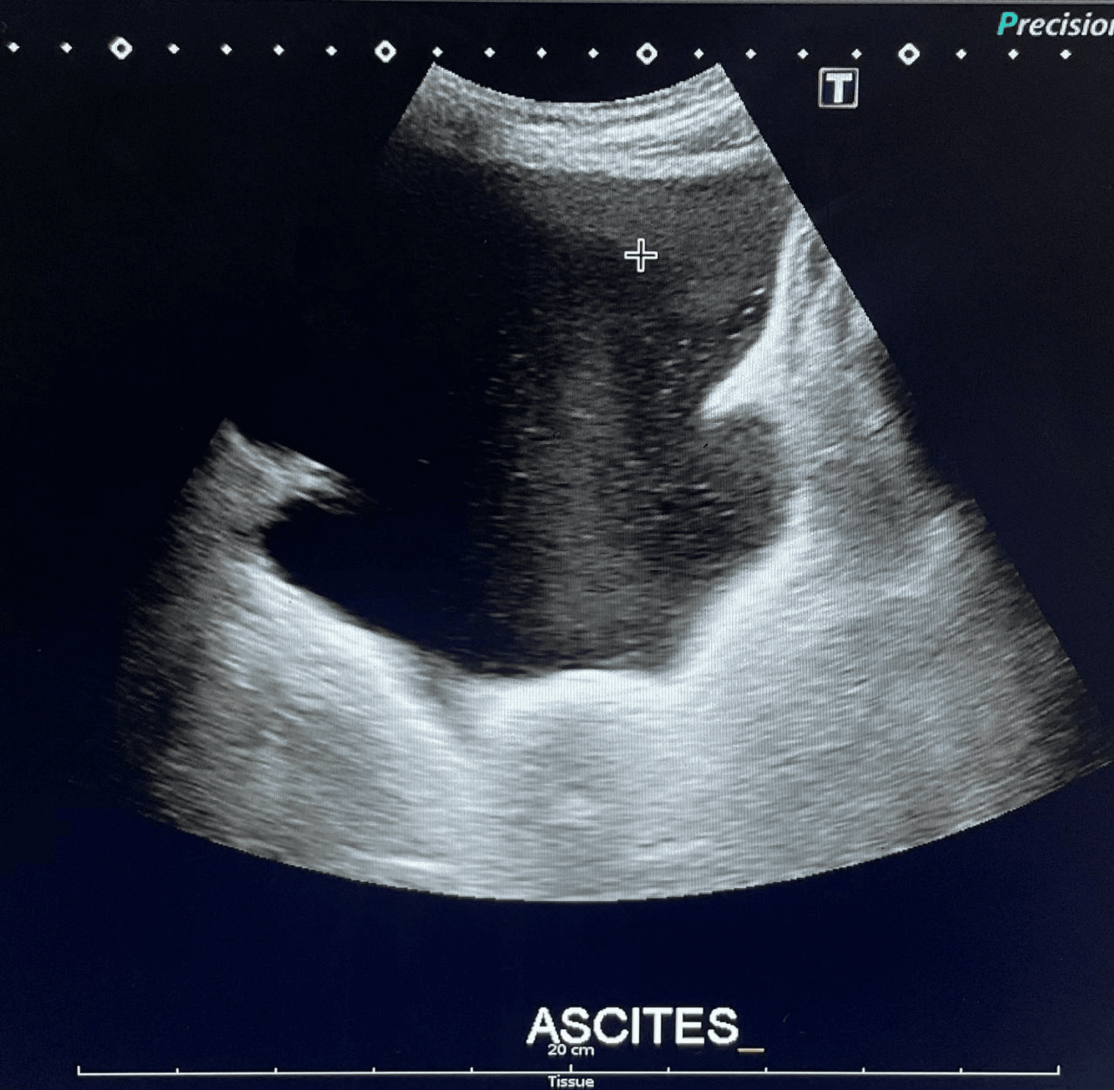
Ultrasound of pelvis revealing gross pelvic ascites with no adnexal masses

Abdominal and pelvic ultrasound detected severe free fluid collection (Figure 2).

Echocardiography showed an ejection fraction of 70% with mild-to-moderate pericardial effusion without pericardial thickening. Initial laboratory findings were obtained. Complete blood count has shown low hemoglobin, low erythrocyte count, normal leukocyte count, and elevated platelet count. Both serum creatinine and urea nitrogen were elevated. Albumin was decreased, and 24-hour urinary protein revealed non-nephrotic range proteinuria. Erythrocyte sedimentation rate was increased. C-reactive protein was slightly elevated, and lactate dehydrogenase was markedly raised. Further investigations were conducted to determine the cause of ascites. Liver enzymes have shown normal levels of aspartate aminotransferase and alanine aminotransferase with elevated alkaline phosphatase (Table 1).

**Table 1:** Laboratory findings of the patient during hospitalization

Laboratory test	Patient’s values	Reference range
Hemoglobin (g/dL)	11.4	Male: 13.5-17.5; Female: 12-16
Erythrocyte count (million/mm^3^)	3.3	Male: 4.3-5.9; Female: 3.5-5.5
Leukocyte count (/mm^3^)	8000	4500-11,000
Platelet count (/mm^3^)	478,000	150,000-400,000
Creatinine (mg/dL)	1.4	0.6-1.2
Urea nitrogen (mg/dL)	98	7-18
Albumin (g/dL)	3	3.5-5.5
24-hour urinary protein (mg/24 h)	626	<150
C-reactive protein (mg/L)	24	<9
Lactate dehydrogenase (U/L)	464	45-200
Aspartate aminotransferase (U/L)	19	12-38
Alanine aminotransferase (U/L)	21	10-40
Alkaline phosphatase (U/L)	244	25-100
Erythrocyte sedimentation rate (mm/h)	90	Male: 0-15; Female: 0-20
Serum-ascites albumin gradient (g/dL)	0.9	Low: <1.1; High: ≥1.1
Ascitic leukocyte count (/μL)	400	<300
CA-125 (U/mL)	198	0-35

**Figure 3:**
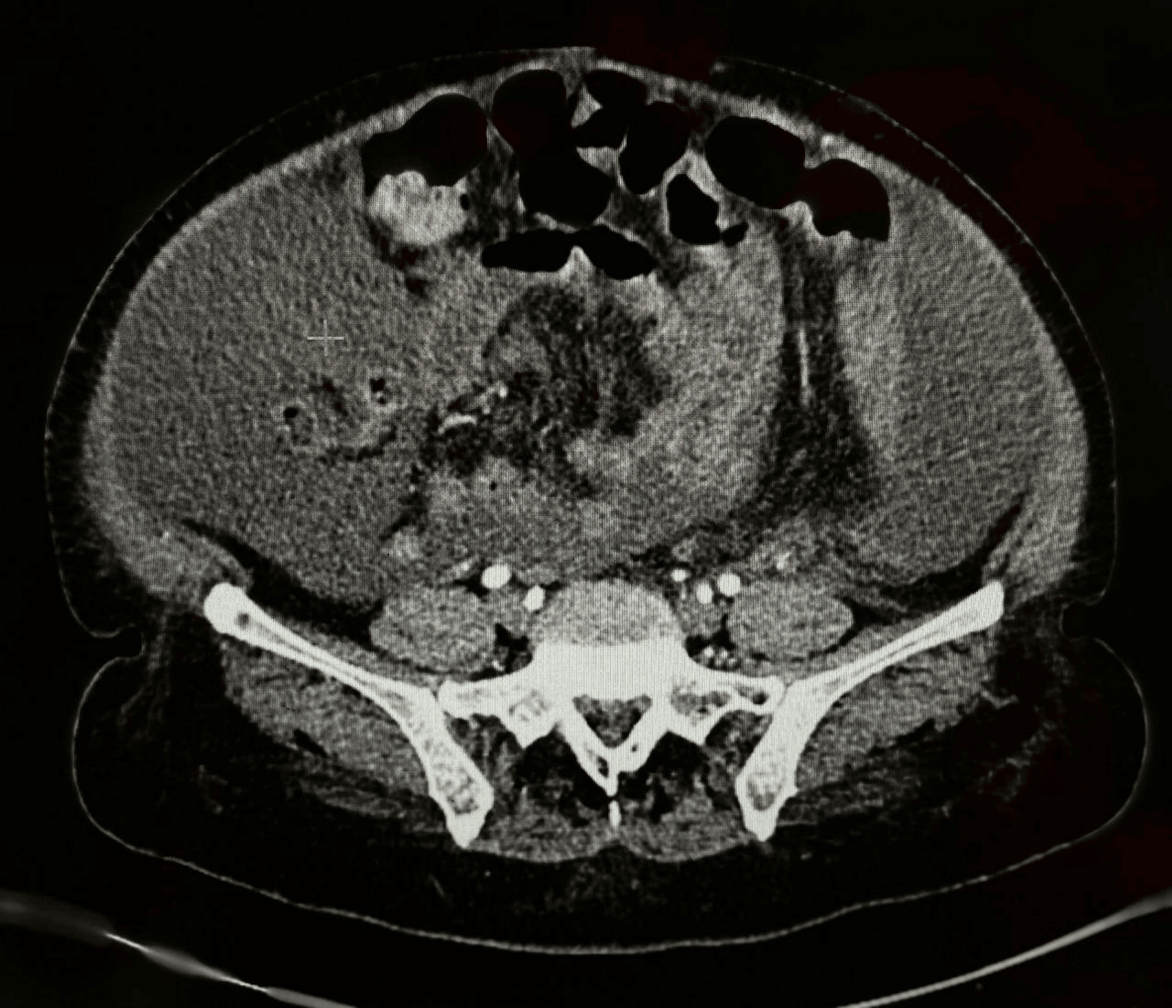
CT scan of the abdomen and pelvis revealing massive ascites without evidence of cancerous lesions

By performing imaging studies, Duplex ultrasonography for hepatic and renal veins revealed normal vasculature with patent and normal blood flow. Therefore, lupus peritonitis, nephrotic syndrome, constrictive pericarditis, Budd-Chiari syndrome, and renal vein thrombosis were ruled out. The patient was started on an intravenous furosemide bolus of 40 mg followed by an intravenous infusion of 20 mg of furosemide for one hour for two days, but without improvement in ascites. Subsequently, an ultrasound-guided paracentesis collected a 400 mL sample of ascitic fluid. Analysis revealed an elevated ascitic leukocyte count with lymphocytic predominance. The serum-ascites albumin gradient (SAAG) was decreased, indicating an exudative etiology of the ascites. As a result, malignant etiology was suspected, and workup revealed an elevated CA-125. However, alpha-fetoprotein and carcinoembryonic antigen were negative. To exclude an ovarian tumor, the patient underwent a CT scan of the abdomen and pelvis, which revealed no cancerous lesions (Figure 3).

Assessment of ascitic fluid culture and tuberculosis polymerase chain reaction yielded no evidence of infection. As a result, the patient was diagnosed with PPMS. Intravenous furosemide was stopped, and 500 mg of intravenous methylprednisolone infusion was initiated daily for three days, with a gradual improvement in symptoms. The patient was then discharged on 50 mg of oral prednisone per day for one month. She presented a month later to the office for follow-up, showing marked improvement in symptoms. Repeated investigations revealed no evidence of ascites or pleural effusion.

## Discussion

We review a case of an SLE patient who presented with a constellation of ascites, pleural effusion, and elevated CA-125. This abnormal presentation is consistent with the rare diagnosis of PPMS. Meigs syndrome is defined as a triad of ascites, pleural effusion, and accompanying benign ovarian tumors. Ascites and pleural effusions could go away once the ovarian tumor is removed. Pseudo-Meigs syndrome includes ascites and pleural effusion, but it is linked to a malignant ovarian tumor. Ascites, pleural effusion, and high CA-125 are also present in PPMS with no connection to either benign or malignant tumors. Pericardial and pleural effusions are common in SLE patients. However, massive ascites without any further complications is rare in SLE patients. For that reason, it is necessary to rule out several etiologies, including nephrotic syndrome, protein-losing enteropathy, Budd-Chiari syndrome, and constrictive pericarditis, to make a diagnosis of PPMS. Echocardiography demonstrated a normal ejection fraction with a normal pericardial thickness. Abdominal ultrasound revealed patent hepatic veins without evidence of thrombosis. In addition, the patient had an exudative pattern of SAAG and a non-nephrotic range proteinuria without pitting edema, which in turn ruled out these diagnoses. The main causes of exudative SAAG patterns are tuberculosis and malignancy. Tuberculosis workup was negative, and no tumor cells were found in the ascitic fluid. Thus, the diagnosis was favorable for PPMS. 

The pathophysiology of effusion in PPMS is still unclear. However, it is hypothesized that it is due to inflammatory pathology affecting the serosa. Autoantibodies produced by self-reacting B lymphocytes bind to circulating antigens, forming immune complexes that deposit on the peritoneum, triggering inflammation and vasculitis in the peritoneal vessels [4]. Type I interferon in SLE produces immune dysregulation. Activated B lymphocytes by immune dysregulation in SLE produce proinflammatory mediators interleukin 6 and tumor necrosis factor, contributing to serosal inflammation [5]. Hyperferritinemia in some patients with PPMS enhances the possibilities of this inflammatory theory [6], and ferritin levels were correlated with SLE disease activity [7,8]. CA-125 is a glycoprotein which was associated with ovarian cancer. However, CA-125 is expressed in mesothelial cells of serosal tissue, and in the epithelium of the fallopian tubes, endometrium, endocervix, lung, breast, prostate and conjunctiva [9,10], so CA-125 can be elevated in many other non-malignant diseases such as tuberculosis, interstitial lung diseases, nephrotic syndrome, endometriosis, SLE, rheumatoid arthritis, and non-gynecological malignancies [6,10,11], thus giving it low sensitivity and specificity in diagnosis of ovarian cancer.

The inflammatory theory can also explain the elevation of CA-125 in PPMS. CA-125 expression is increased when the mesothelial cells are activated through inflammatory cytokines, such as vascular endothelial growth factor (VEGF), fibroblast growth factor, interleukin-1b, and interferon-gamma. [6,12]. Raised levels of these cytokines during an SLE flare-up activate mesothelial cells and elevate CA-125, which also induces fluid leakage and third-space fluid accumulation. The role of inflammatory cytokines in increasing CA-125 levels is supported by the findings of Ataseven et al., who measured the level of CA-125 in inflammatory bowel disease patients and compared them to a control group. They resulted in a significant increase in CA-125 level in patients with ulcerative colitis, but a non-significant increase in patients with Crohn’s disease [10].

Another theory regarding the relationship between CA-125 glycoprotein secretion and ascites formation was proposed by Basaran et al. He hypothesized that the CA-125 level can increase due to the ascites itself, as it irritates the mesothelial cells, stimulating the production of CA-125 [11]. This hypothesis was supported by Sevinc et al., who concluded that serum CA-125 levels were elevated in patients with nephrotic syndrome and ascites, without any other causes of elevated CA-125 [13]. Therefore, we can conclude that CA-125 elevation in PPMS patients results from the activation of mesothelial cells by both inflammatory cytokines and irritation from ascitic fluid, with autoantibodies triggering these two activation mechanisms.

The causes of abdominal serositis or related gastrointestinal involvement in SLE are still unknown, but they tend to occur during lupus flares and coincide with systemic disease activity [14,15]. The triggers for these flares are not fully understood. However, some possible triggers were mentioned in previous cases. McVorran et al. suggested that stress from the recent surgery triggered an SLE flare-up and PPMS [12]. Additional cases involved lupus nephritis and renal impairment, indicated by the kidney biopsy and blood urea nitrogen levels, which were identified as potential triggers for PPMS [6,16,17].

Another study has shown that leflunomide therapy can induce the development of PPMS by its effect on cytokine levels and Th1/Th2 ratio [18]. Furthermore, Asghar et al. asserted that patients may develop PPMS after SLE flare triggered by pregnancy [19].

## Conclusions

We report a case of a patient with SLE who presented with a constellation of ascites, pleural effusion, and high CA-125. It is possible that this atypical appearance is congruent with the extremely uncommon diagnosis of PPMS. PPMS is a diagnosis that is based on exclusion, and the report provided clarification regarding the method of diagnosis. In any patient with SLE diagnosed with extensive ascites, a differential diagnosis for PPMS should be considered once malignancies have been ruled out. This is done in order to avoid conducting unnecessary and intrusive investigations. Because polyserositis in PPMS is associated with a flare-up of SLE, the patient was treated with standard immunosuppressive therapy and had a positive response in terms of renal function, ascites, pleural effusion, and pericardial effusion.
